# NRF2-HIF2α Signaling Attenuates Endothelial Cell Senescence and Maintains Intercellular Junctions in Diabetes

**DOI:** 10.7150/ijbs.96719

**Published:** 2024-07-22

**Authors:** Jian Shen, Yifan Lai, Yaner Lu, Yabin Liu, Jinlong Zhang, Yan Wu, Yunan Pan, Haibo Chen, Qiyue Gao, Qucheng Wei, Yuwen Chen, Jian Ye, Yinuo Lin, Bingchen Liu, Jun Jiang, Jinliang Nan

**Affiliations:** 1 Department of Cardiology of The Second Affiliated Hospital, School of Medicine, Zhejiang University, Hangzhou, China.; 2 State Key Laboratory of Transvascular Implantation Devices, Hangzhou 310009, China.; 3 Cardiovascular Key Laboratory of Zhejiang Province, Hangzhou 310009, China.; 4 Research Center for Life Science and Human Health, Binjiang Institute of Zhejiang University, Hangzhou 310053, China.; 5 Wenzhou Municipal Key Cardiovascular Research Laboratory, Department of Cardiology, The First Affiliated Hospital, Wenzhou Medical University, Wenzhou 325000, China.

**Keywords:** NRF2, HIF-2α, diabetes, senescence, intercellular junctions, Oltipraz

## Abstract

In the context of diabetes, endothelial cells frequently exhibit compromised intercellular junctions and accelerated cellular senescence simultaneously. The precise mechanisms underlying these issues and the identification of effective treatments remain largely undefined. Our findings reveal that human umbilical vein endothelial cells (HUVECs) can counteract senescence and uphold the integrity of intercellular junctions under mildly to moderately elevated glucose levels (10 mM and 15 mM) via two primary mechanisms: i) The acetylation of NRF2 at lysine residues K56, K68, and K52 prevents its ubiquitination, enhancing the transcription of antioxidant genes GST, SOD1, and GPX1. This activity diminishes cytoplasmic oxidative stress, thereby mitigating endothelial cell senescence. ii) The interaction between the Neh2 domain of NRF2 and the PAS-B domain of HIF-2α within the nucleus curtails the attachment of HIF-2α to the NOX4/p22phox promoter. This action lessens oxidative stress near the cell membrane, maintaining intercellular junctions by safeguarding the disulfide bonds in occludin and E-cadherin from disruption. However, these protective strategies prove insufficient under severe hyperglycemic conditions (25 mM). Further investigation has identified Oltipraz, an activator of NRF2, as also promoting the degradation of HIF-2α. Through its simultaneous modulation of NRF2 and HIF-2α, Oltipraz significantly reduces cellular senescence and prevents the deterioration of intercellular junctions in HUVECs subjected to high glucose concentrations (25 mM). Our research positions Oltipraz as a promising therapeutic candidate for mitigating diabetes-induced vascular endothelial damage, potentially offering benefits against diabetes-related atherosclerosis and valvular calcification.

## Introduction

Diabetes mellitus represents a worldwide epidemic, impacting millions globally. Vascular complications associated with diabetes contribute significantly to the morbidity and mortality of affected individuals^1^. The endothelium, which lines the interior surface of blood vessels, consists of endothelial cells. These cells are pivotal in managing vascular tone, permeability, and the process of angiogenesis^2^. Endothelial cell dysfunction plays a central role in the development of diabetic vascular complications, which are characterized by cellular senescence and weakened intercellular junctions^3^, ^4^.

Cellular senescence and compromised intercellular junctions are common hallmarks across various diseases^5^, ^6^. In Alzheimer's disease, the senescence of cells is marked by diminished vascular cell junctions, leading to increased blood-brain barrier permeability^4^, ^7^, ^8^. Similarly, in ovarian cancer, senescence-associated degradation of intercellular junctions in the peritoneal mesothelium promotes the invasion of cancer cells across the mesothelial layer^9^. Hyperglycemia has been shown to induce endothelial cell senescence and disrupt intercellular junctions, leading to dysfunction in the vascular endothelium. Zheng *et al.* found that human umbilical vein endothelial cells from women with hyperglycemia in pregnancy exhibited excessive senescence, with increased expression of senescence markers^10^. Hashmat *et al.* demonstrated that hyperglycemia impaired blood-brain barrier integrity through its effects on endothelial cell characteristics and function^11^. These studies suggest that hyperglycemia can promote endothelial cell senescence and disrupt intercellular junctions, resulting in dysfunction of the vascular endothelium. However, the precise molecular mechanisms that drive the concurrent changes in cellular senescence and intercellular junctions remain poorly understood. Therefore, further research is crucial to decipher the intricate relationship between cellular senescence and intercellular junctions, to discover new therapeutic targets.

As a metabolic disorder, diabetes triggers oxidative stress by disrupting the balance between the production of reactive oxygen species and antioxidant defenses. Sustained high glucose concentrations exacerbate oxidative stress in endothelial cells, accelerate cellular senescence, and compromise the integrity of intercellular tight junctions, as evidenced by decreased levels of junction proteins such as occludin and E-cadherin^12^, ^13^. Consequently, these observations suggest that oxidative stress may be the underlying link between cellular senescence and alterations in intercellular junctions due to diabetes. This damage cascade can intensify, leading to lipid peroxidation and deterioration of vascular endothelial barrier function, potentially culminating in the development of atherosclerosis^13^, ^14^.

In the diabetic milieu, endothelial cells confront elevated oxidative stress, which triggers endothelial dysfunction and increases the risk of diabetic complications. NRF2 plays a pivotal role in defense and activates antioxidant genes such as SOD, CAT, and GSH, which mitigate the production of cytoplasmic reactive oxygen species (ROS)^15^, ^16^. In contrast, the hypoxia-inducible factor (HIF)-2α plays a complex role in the oxidative stress landscape^17^. On one front, it mirrors NRF2's antioxidant capacity by diminishing cytoplasmic ROS levels^18^. Conversely, HIF-2α can also enhance the expression of genes associated with NADPH oxidases, potentially increasing oxidative stress adjacent to the cell membrane^19^. Despite these insights, the intricate regulatory interplay between NRF2 and HIF-2α within diabetic endothelial cells warrants further exploration to fully unravel.

This research revealed that endothelial cells possess an innate defense mechanism against damage caused by diabetes. A modest elevation in glucose concentrations triggers the activation of NRF2 via acetylation, safeguarding against cellular senescence and sustaining the integrity of intercellular junctions. Specifically, NRF2 alleviates oxidative stress in the cytoplasm to avert cellular senescence and prevents HIF-2α from causing oxidative damage near cell membranes, thereby maintaining the cohesion of cell junctions. Nonetheless, elevated glucose levels can override this protective measure. We identified Oltipraz, a pharmaceutical that amplifies NRF2 activity and curtails HIF-2α, bolstering the defense of diabetic endothelial cells against senescence and the weakening of cell junctions.

## Results

**Figure 1:**
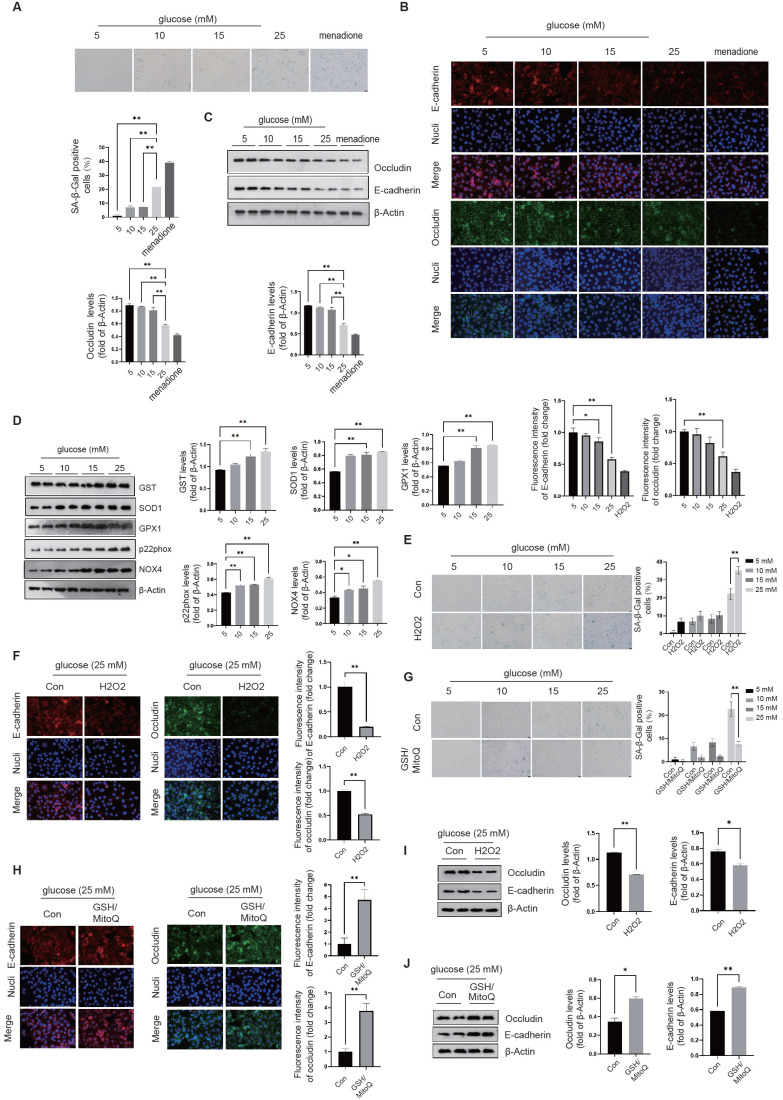
** The oxidative mechanism in diabetic endothelial cells regulates both cellular senescence and intercellular junctions. A,** The activity of β-galactosidase in HUVECs was measured at glucose concentrations of 5, 10, 15, and 25 mM, with menadione-treated groups serving as positive controls. **B&C,** The tight junction proteins occludin and E-cadherin in HUVECs exposed to different glucose concentrations (5 mM, 10 mM, 15 mM, and 25 mM) were measured using immunofluorescence staining and Western blot analysis. **D,** Protein levels of GST, SOD1, GPX1, p22phox, and NOX4 in HUVECs were quantified following treatment with different glucose concentrations (5 mM, 10 mM, 15 mM, and 25 mM). **E,** β-Galactosidase activities were assessed in HUVECs after exposure to 100 µM H2O2 or control conditions (Con) across varying glucose concentrations (5 mM, 10 mM, 15 mM, and 25 mM). **F,** At a glucose concentration of 25 mM, HUVECs treated as described in E were analyzed for the distribution of occludin and E-cadherin using immunofluorescence staining. **G,** HUVECs treated with GSH/MitoQ (10 mM/5 mM) across different glucose concentrations (5 mM, 10 mM, 15 mM, and 25 mM) were evaluated for β-galactosidase activity. **H,** Following treatment as described in G at a glucose concentration of 25 mM, the distribution of occludin and E-cadherin in HUVECs was determined using immunofluorescence staining. **I&J,** HUVECs treated as outlined in F & H, respectively, had their occludin and E-cadherin protein levels assessed via Western blot.

**The oxidative mechanism in diabetic endothelial cells regulates both cellular senescence and intercellular junctions.** In this study, we investigated the effects of different glucose concentrations (5mM, 10mM, 15mM, and 25mM) on endothelial cellular senescence in *in vitro* conditions. We measured the activity of β-galactosidase, a widely accepted senescence marker, to assess cellular senescence. We found that there was a significant increase in β-galactosidase activity at a glucose concentration of 25 mM, indicating a higher degree of cellular senescence compared to the lower concentration groups (5mM, 10mM, and 15mM glucose) (Fig. 1A). We assessed intercellular junctions by measuring the levels of two key tight junction proteins, occludin and E-cadherin, using immunofluorescence staining and Western blot; We found that at a glucose concentration of 25mM, there was a significant decrease in protein levels of occludin and E-cadherin compared to lower glucose concentrations (5mM, 10mM, and 15mM) (Fig. 1B&C), indicating impaired intercellular junctions associated with senescence. We found that the effects of glucose on senescence are similar to those of the well-established cellular senescence inducer, menadione (Fig. 1B&C), suggesting that high glucose concentration at 25mM can induce endothelial cell senescence and impair intercellular junctions.

We used different le glucose concentrations to mediate oxidative stress responses and found that at concentrations of 15mM and 25mM, there was a significant increase in the levels of glutathione S-transferase (GST), superoxide dismutase 1 (SOD1), and Glutathione Peroxidase 1 (GPX1) proteins (Fig. 1D). Compared to the 5mM glucose condition, protein levels of NADPH oxidase-associated proteins, including p22phox and NOX4, were increased in the higher glucose conditions (Fig. 1D). We added H2O2 to the culture medium to promote oxidative stress and GSH/MitoQ to inhibit oxidative stress. We found that H2O2 further increases cellular senescence (Fig. 1E) and disrupts intercellular junctions at different glucose concentrations (Fig. 1F), while GSH/MitoQ can simultaneously alleviate cellular senescence (Fig. 1G) and maintain intercellular junctions (Fig. 1H). These data further confirm that the endogenous antioxidant stress mechanism in diabetic endothelial cells is crucial for alleviating cellular senescence and maintaining intercellular junctions. We next examined the influence of oxidative stress on tight junctions in diabetic endothelial cells by interfering with the oxidative stress status of the cells and measuring the protein levels of occludin and E-cadherin. Our results showed that H2O2 treatment significantly decreased the protein levels of occludin and E-cadherin (Fig. 1I), whereas GSH/MitoQ intervention significantly increased their protein levels (Fig. 1J).

**Figure 2:**
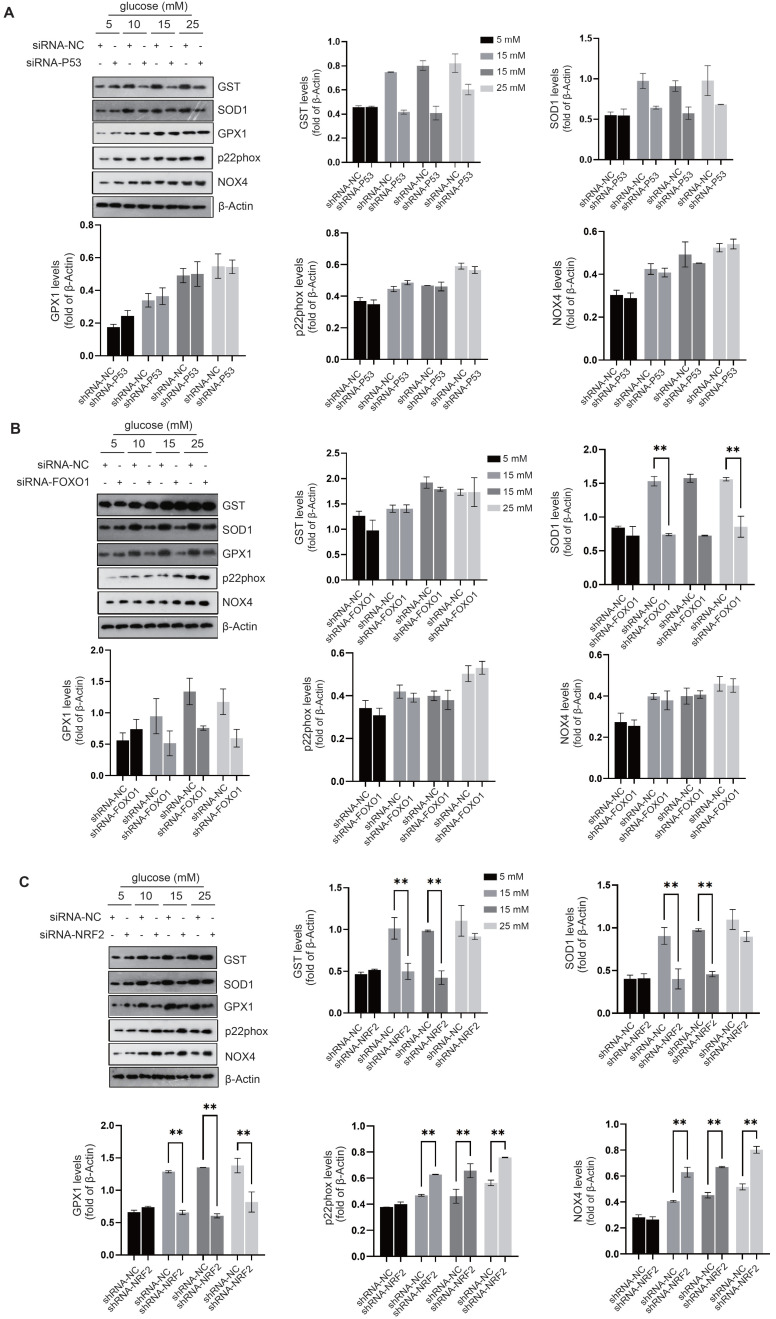
** Activation of NRF2 curtails oxidative stress in the cytoplasm and around the cell membrane. A-C,** Following the knockdown of P53 (A), FOXO1 (B), or NRF2 (C) in HUVECs, the expression levels of oxidative stress-related enzymes, including GST, SOD1, GPX1, p22phox, and NOX4, were quantified using Western blot analysis across varying glucose concentrations (5 mM, 10 mM, 15 mM, and 25 mM).

**Figure 3:**
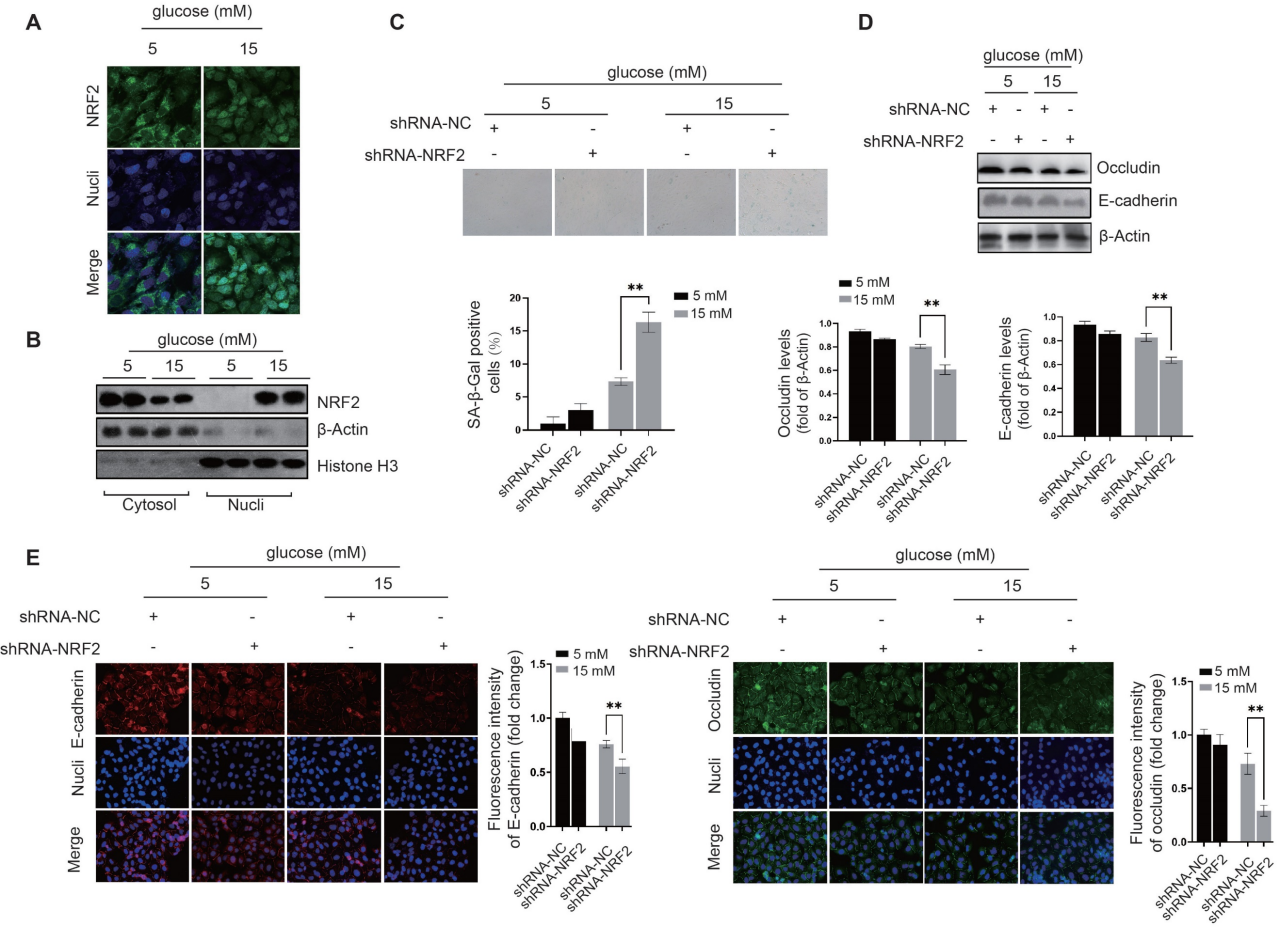
** NRF2 activation prevents cellular senescence and preserves intercellular junctions in diabetic endothelial cells. A,** The nuclear translocation of NRF2 in HUVECs exposed to glucose concentrations of 5 and 15 mM was examined by immunofluorescence staining. **B,** Following the treatment of HUVECs as described, cytoplasmic and nuclear proteins were isolated. Western blot analysis was conducted to quantify NRF2 protein levels in each fraction, with β-actin and histone H3 serving as loading controls for the cytoplasmic and nuclear fractions, respectively. **C,** After transfection with lentivirus-mediated shRNA-NC (non-coding) and shRNA-NRF2, HUVECs were cultured in medium containing either 5 mM or 15 mM glucose. The senescence level of the cells was evaluated by measuring β-galactosidase activity. **D&E,** Under the procedures outlined in C, treated HUVECs were analyzed for the protein levels and distribution of occludin and E-cadherin using Western blot and immunofluorescence staining.

**NRF2 activation prevents cellular senescence and preserves intercellular junctions in diabetic endothelial cells.** We investigated the regulatory effects of transcription factors related to cellular senescence, including P53^20^, FOXO1^21^, and NRF2^22^, to elucidate their roles in modulating the cellular response to oxidative stress. Compared to the control group, siRNA-mediated knockdown of P53 suppressed the expression of GST and SOD1 in diabetic endothelial cells (Fig. 2A); While knockdown of FOXO1 inhibited the expression of SOD1 and GPX1, no significant changes were observed in other antioxidant or pro-oxidant enzymes (Fig. 2B). Compared with the control group, siRNA-mediated knockdown of NRF2 significantly suppressed the increase in SOD1, GST and GPX1 protein levels in diabetic endothelial cells and significantly increased the protein levels of p22phox and NOX4 (Fig. 2C). Previous studies have shown that occludin and E-cadherin are mainly regulated by NADPH oxidases (NOX)^23^. Therefore, we hypothesize that NRF2 in diabetic endothelial cells alleviates cellular senescence by regulating antioxidant enzymes such as SOD1, GST, and GPX1 and reducing the disruption of intercellular junctions by modulating p22phox and NOX4. NRF2 is a transcription factor that regulates the response to oxidative stress primarily by binding to the antioxidant response element (ARE)^16^. The ARE is a sequence in the promoter region of antioxidant response genes that responds to oxidative stress signals and thereby initiates transcription of these target genes. Using immunofluorescence staining, we also found that elevated glucose levels cause a significant increase in NRF2 nuclear translocation in HUVECs (Fig. 3A), indicating the activation state of NRF2. The protein levels of NRF2 were further detected after separation of cytoplasm and nucleus, confirming that under high glucose conditions, nuclear retention of NRF2 was significantly increased in HUVECs (Fig. 3B). To further elucidate the precise role of NRF2, we used lentivirus-mediated shRNA targeting NRF2 to achieve its knockdown. Remarkably, our experiments showed that even in a slightly to moderately elevated glucose environment (5 mM and 15 mM), significant endothelial cell senescence (Fig. 3C) and the appearance of abnormal intercellular junctions (Fig. 3D&E) were observed in HUVECs with NRF2 knockdown. The above data suggest that endothelial cells activate NRF2 as a protective mechanism in response to higher glucose conditions. This activation helps to alleviate cellular senescence and maintain intercellular junctions.

**Figure 4:**
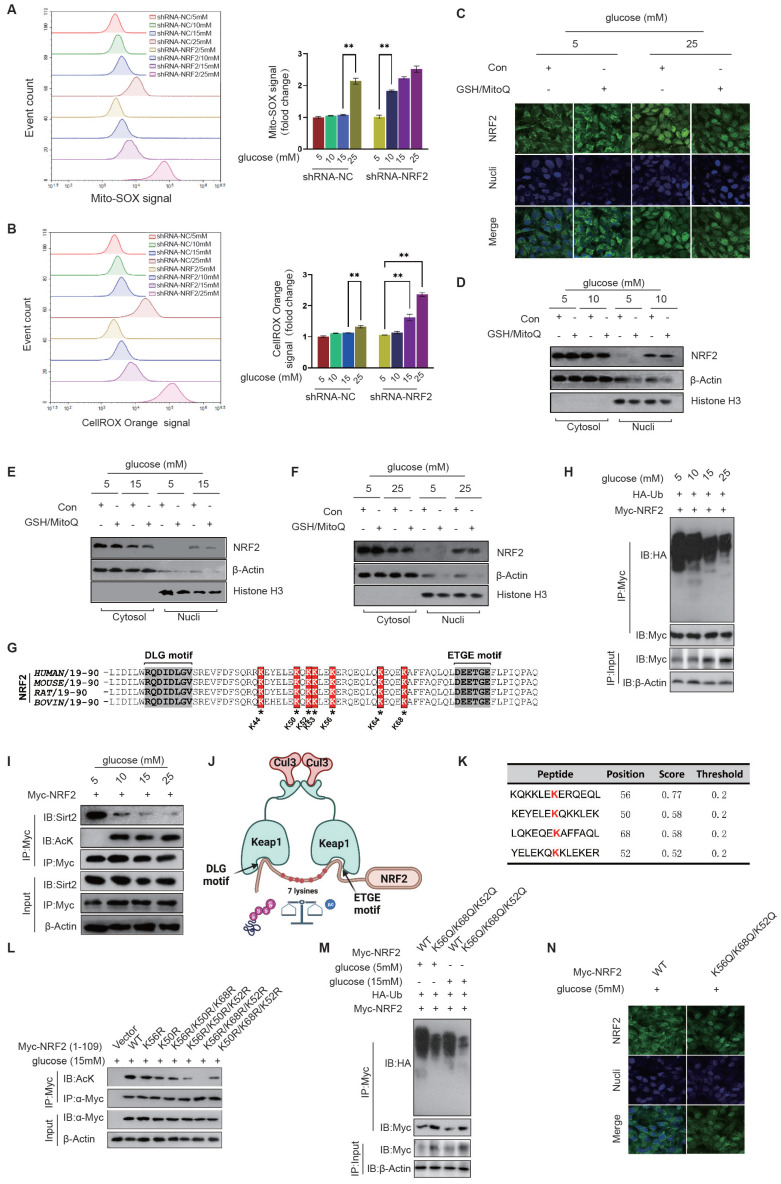
** Acetylation of NRF2 in diabetic endothelial cells promotes its activation by inhibiting its ubiquitination independent of oxidative stress status. A&B,** HUVECs exposed to various glucose concentrations (5 mM, 10 mM, 15 mM, and 25 mM) were treated with shRNA to either inhibit NRF2 expression or not. ROS levels were measured using flow cytometry by detecting signals from MitoSOX, which marks mitochondrial oxidative stress (A), and CellROX Orange, which indicates cytoplasmic oxidative stress (B). **C,** Following GSH/MitoQ treatments, HUVECs exposed to 5 mM or 25 mM glucose concentrations underwent immunofluorescence staining to observe NRF2's subcellular localization. **D-F,** Post GSH/MitoQ treatment and exposure to 10 mM, 15 mM, and 25 mM glucose concentrations, HUVECs had their cytoplasmic and nuclear proteins separated. Western blot analysis was utilized to assess NRF2 protein levels. **G,** The conservation of ETGE and DLG motifs along with key lysine residues was explored through cross-species comparison. **H,** NRF2 ubiquitination levels were evaluated in HUVECs cultured at glucose concentrations of 5 mM, 10 mM, 15 mM, and 25 mM. **I,** HUVECs treated as described in B were assessed for NRF2 acetylation. The interaction between Sirt2 and NRF2 was also explored using co-IP assays. **J,** A schematic diagram illustrates the interactions between Keap1 and NRF2, highlighting the OLG and ETGE motifs, and seven lysine residues (K44, K50, K52, K53, K56, K64, and K68) involved in ubiquitination and acetylation. The diagram also shows the competitive inhibition between NRF2's acetylation and ubiquitination, emphasizing the influence of the cellular environment on these processes. **K,** The GPS-PAIL tool identified lysine acetylation sites on NRF2, with K56, K50, K68, and K52 being the highest-ranked potential sites. **L,** Myc-NRF2(1-109) mutants were expressed in HUVECs as indicated, followed by immunoprecipitation and anti-acetyl-lysine antibody-based Western blot. **M,** The effect of the NRF2-K56Q/K68Q/K52Q mutation on NRF2 ubiquitination was investigated in HUVECs at glucose concentrations of 5 mM and 15 mM, using immunoprecipitation and Western blot to detect NRF2 ubiquitination. **N,** HUVECs were categorized into two groups, NRF2-WT and NRF2-K56Q/K68Q/K52Q, and cultured in medium containing either 5 mM or 15 mM glucose. The subcellular localization of NRF2 was determined using immunofluorescence staining.

**Activation of NRF2 in diabetic endothelial cells is independent of oxidative stress status.** The Keap1-NRF2 signaling pathway is a cellular antioxidant defense mechanism involved in the regulation of cellular redox balance, alleviation of oxidative stress and other processes^16^. Oxidative stress can lead to an increase in intracellular oxidants, which activates NRF2 and allows it to dissociate from Keap1, enter the nucleus and promote the expression of antioxidant genes, thereby increasing cellular antioxidant capacity and protecting cells. Activation of NRF2 by Keap1 is associated with the reduced ubiquitination and degradation of NRF2^24^. However, under conditions of mild (10mM) and moderate (15mM) glucose elevation, although significant activation of NRF2 was observed, there was no significant increase in mitochondrial and cytoplasmic ROS levels as indicated by the intensity of MitoSOX and CellROX Orange fluorescence (Fig. 4A&B). When NRF2 expression was inhibited using shRNA, mild (10mM) and moderate (15mM) glucose elevation induced high-intensity MitoSOX and CellROX-Orange signals (Fig. 4A&B), suggesting that NRF2 activation is a key mechanism for endothelial cells to relieve oxidative stress.

We next examined whether the activation and nuclear translocation of NRF2 in diabetic endothelial cells depends on oxidative stress. Despite inhibiting cytoplasmic ROS production with GSH and mitochondrial ROS production with MitoQ, we still observed nuclear translocation of NRF2 at a glucose concentration of 25mM (Fig. 4C). Furthermore, by separating the cell fraction from cytoplasm and nucleus, we found that GSH and MitoQ treatment did not reduce the nuclear retention of NRF2 in HUVECs at glucose concentrations of 10 mM, 15 mM, and 25 mM (Fig. 4D-F). These data suggest that in mildly to moderately elevated glucose environments, activation, nuclear translocation, and retention of NRF2 in diabetic endothelial cells are not dependent on oxidative stress.

**Acetylation of NRF2 in diabetic endothelial cells promotes its activation by inhibiting its ubiquitination.** Previous literature has reported that Keap1 identifies and binds to the DLG and ETGE motifs in NRF2, thereby promoting protein degradation of NRF2 through ubiquitination modification of the lysine residues between the two motifs^24^. The domains containing DLG and ETGE are highly conserved across species (Fig. 4G). Studies have shown that NRF2 is ubiquitinated and degraded in the cytoplasm under physiological conditions, resulting in limited localization in the cytoplasm. However, under pathological stimulation, ubiquitination and degradation of NRF2 decrease and it translocates to the nucleus to exert its cellular protective functions. Compared to the 5mM glucose environment, HUVECs showed a significant reduction in NRF2 ubiquitination modification under 10mM, 15mM, and 25mM glucose concentrations, with 15mM and 25mM concentrations showing more significant reductions (Fig. 4H). Our preliminary data suggest that activation of non-oxidative stress-dependent NRF2 occurs in diabetic endothelial cells, and we speculate that other post-translational modifications of NRF2 may regulate its ubiquitination modification in these cells. We also observed a significant increase in the acetylation modification of NRF2 under high glucose treatment and a corresponding reduction in the interaction between Sirt2 and NRF2 (Fig. 4I). These data suggest that reduced Sirt2 function in diabetic endothelial cells promotes the increase in NRF2 acetylation modification.

As shown in the schematic diagram, Keap1 recognizes the DLG and ETGE motifs of NRF2 and mediates the ubiquitination modification of seven lysine residues between them, including K44, K50, K52, K53, K56, K64, and K68; When the deacetylation function of Sirt2 is reduced, it can lead to an increase in the acetylation modification of these lysine residues, thereby inhibiting the ubiquitination modification at the corresponding sites; The acetylation and ubiquitination modifications of NRF2 can competitively inhibit each other and can be adjusted according to the cellular environment (Fig. 4J). The GPS-PAIL tool was used to identify lysine acetylation sites on NRF2, with scores ranked from highest to lowest value. The results showed that K56, K50, K68, and K52 were most likely sites for acetylation modification (Fig. 4K). According to the score ranking of GPS-PAIL, we mutated the most likely combinations of acetylation sites (K-R, lysine was mutated to arginine to simulate non-acetylate mutations), including K56R, K50R, K56R/K50R/K68R, K56R/K50R/K52R, K56R/K68R/K52R, and K50R/K68R/K52R. We expressed the amino acid sequence 1-109 of Myc-NRF2 with the above-mentioned mutations (containing DLG and ETGE motifs and only these seven lysines) to prevent any effects of other lysine residues on NRF2. We then immunoprecipitated each Myc-NRF2(1-109) mutant and used anti-acetyl-lysine antibodies for detection. The results showed that K56, K68, and K52 are acetylation modification sites of NRF2 in diabetic endothelial cells (Fig. 4L). We created the NRF2 mutation K56Q/K68Q/K52Q by replacing the corresponding lysines with glutamine to simulate an acetylation modification. We found that the NRF2-K56Q/K68Q/K52Q mutation significantly inhibited the ubiquitination modification of NRF2 in HUVECs at both 5mM and 15mM glucose concentrations, suggesting that the acetylation modification competitively inhibits the ubiquitination modification of NRF2 in diabetic endothelial cells (Fig. 4M). Furthermore, we used immunofluorescence staining to show that the NRF2-K56Q/K68Q/K52Q mutant exhibited significant nuclear translocation in HUVECs even at a glucose concentration of 5 mM (Fig. 4N), suggesting that K56, K68, and K52 in NRF2 modified by acetylation are crucial for its activation in diabetic endothelial cells.

**The interaction between NRF2 and HIF-2α increases their nuclear retention capacity in diabetic endothelial cells**. Studies have shown that NRF2 is activated under oxidative stress and binds to antioxidant response elements (ARE) to promote the transcriptional expression of genes such as GST, SOD1, and GPX1^15^. Nevertheless, NRF2 does not directly control NADPH oxidase transcriptionally. Figure 2A shows that NRF2 inhibits the expression of NADPH oxidase-related subunits in diabetic endothelial cells. Based on these findings, we speculate that NRF2, together with other transcription factors, may repress the expression of NADPH oxidase-related subunit genes.

**Figure 5:**
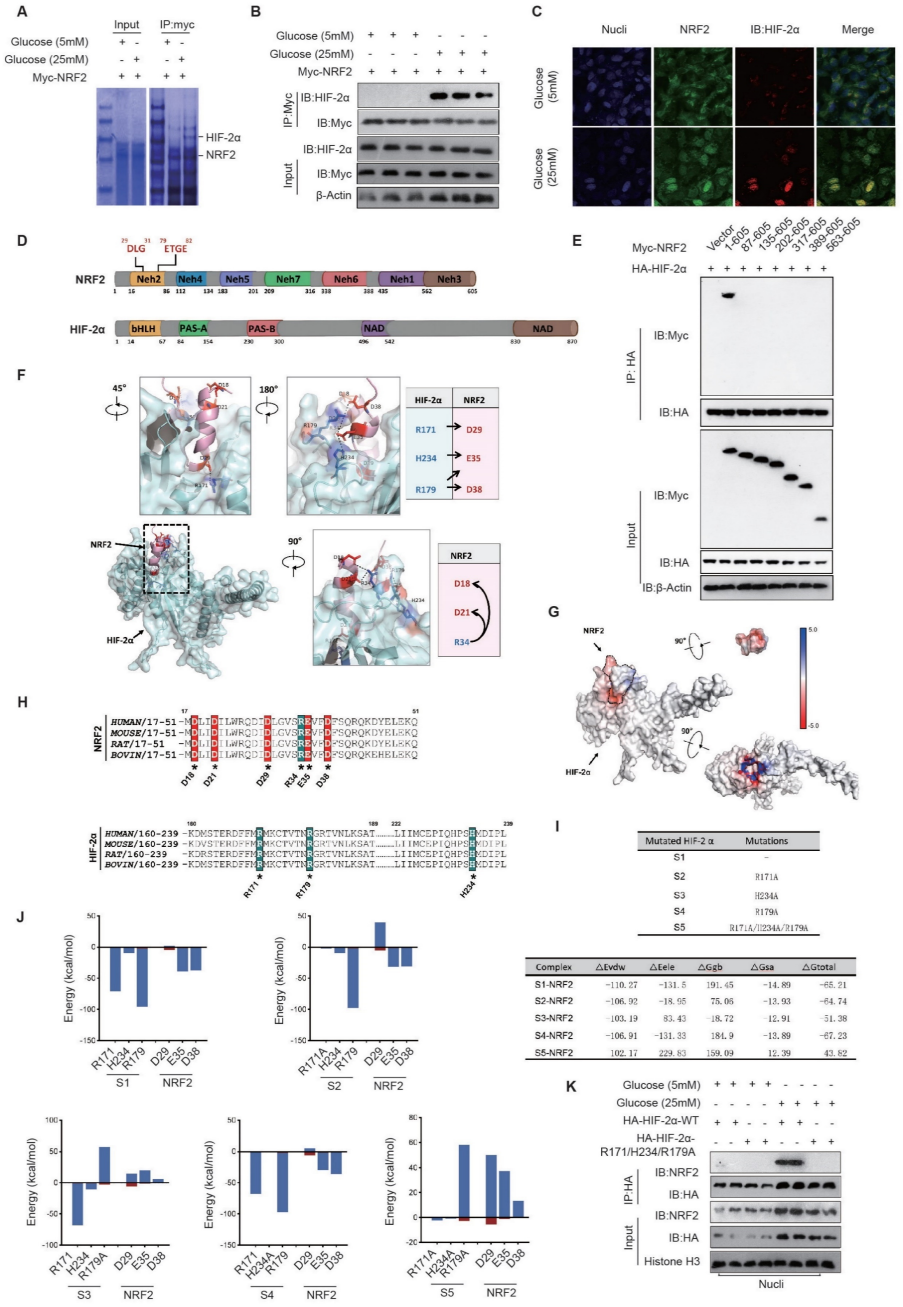
** The electrostatic interaction between NRF2-Neh2 and HIF-2α-PAS-B in diabetic endothelial cells promotes their nuclear retention. A,** The proteins interacting with NRF2 in HUVECs at glucose concentrations of 5 mM and 25 mM were identified through immunoprecipitation and Coomassie Brilliant Blue staining. **B,** At glucose concentrations of 5 mM and 25 mM, HUVECs underwent NRF2 immunoprecipitation using an anti-Myc antibody, with the NRF2-HIF-2α interaction being detected via Western blot. **C,** The nuclear colocalization of NRF2 and HIF-2α in HUVECs, treated with glucose concentrations of 5 mM and 25 mM, was visualized using immunofluorescence staining. **D,** The schematic diagram illustrates the domain structures of NRF2 and HIF-2α. HIF-2α is composed of two Per-Arnt-Sim (PAS) domains (PAS-A and PAS-B), an N-terminal activation domain (NAD), a basic helix-loop-helix (bHLH), and a C-terminal domain (CAD). In contrast, NRF2 features seven distinct domains, labeled Neh2 through Neh7. **E,** In HEK293T cells, various FLAG-tagged NRF2 truncations and HA-tagged HIF-2α were expressed, followed by immunoprecipitation using an anti-HA antibody to identify their interactions through Western blot. **F,** ZDOCK docking analysis helped determine the most probable docking configuration between NRF2 and HIF-2α, with the primary interaction mode being selected based on the docking score. The analysis focused on the PAS-B domain of HIF-2α and the DLG motif in the Neh2 domain of NRF2, visualizing electrostatic interactions between specific amino acid residues. **G,** The APBS method calculated the electrostatic potential distribution on the surfaces of NRF2-Neh2 and HIF-2α-PAS-B structures. **H,** Positively and negatively charged amino acids at the docking interface were identified across species using amino acid sequence alignment analysis. **I,** Energy decomposition was conducted for the five docking models as described in the tables. **J,** Bar graphs illustrate the energy contributions of residues to the electrostatic interactions between HIF-2α-PAS-B and NRF2-Neh2. **K,** Following the expression of HA-HIF-2α-WT or HA-HIF-2α-R171A/H234A/R179A in HUVECs exposed to medium containing either 5 mM or 25 mM glucose, a co-IP assay was conducted to assess their interaction with NRF2 in the nucleus.

To identify potential transcription factors that interact with NRF2 in diabetic endothelial cells, we used anti-NRF2 antibodies for NRF2 immunoprecipitation and analyzed the variations in NRF2-associated proteins between endothelial cells exposed to 5 mM and 25 mM glucose environments by Coomassie Brilliant Blue staining. Together with the results of protein mass spectrometry analysis, we discovered that the interaction between NRF2 and HIF-2α was enhanced by a 25 mM glucose concentration (Fig. 5A). The co-IP assay confirmed the presence of a high glucose environment (25mM) mediating the interaction between NRF2 and HIF-2α in HUVECs (Fig. 5B). The nuclear colocalization of NRF2 and HIF-2α was significantly promoted by a high concentration glucose environment (25 mM) compared to the normal glucose concentration (5 mM), as shown by immunofluorescence staining (Fig. 5C). These results collectively suggest the nuclear translocation of NRF2 and its interaction with HIF-2α in diabetic endothelial cells.

Considering the role of HIF-2 in controlling transcription of both SOD genes and NADPH oxidase-related genes^19^, along with the evidence from Fig. 2A indicating the ability of NRF2 to control the expression of NADPH-associated proteins such as p22phox and NOX4 in diabetic endothelial cells, we suggest that the interaction between NRF2 and HIF-2 could inhibit the binding of HIF-2 to the promoters of genes associated with NADPH oxidase. This interaction potentially promotes a synergistic increase in the expression of antioxidant genes, particularly those encoding SOD.

**The electrostatic interaction between NRF2-Neh2 and HIF-2α-PAS-B in diabetic endothelial cells promotes their nuclear retention.** NRF2 comprises seven domains with diverse roles, such as interacting with Keap1 (Neh2), mediating interactions with transcriptional co-factors (Neh4), interacting with chromatin remodeling complexes (Neh5), regulating transcriptional activity (Neh7), controlling degradation (Neh6), and activating transcription (Neh3)^15^. HIF-2α is composed of multiple domains, including basic helix-loop-helix (bHLH), two Per-Arnt-Sim (PAS) domains named PAS-A and PAS-B, and two functionally important regions called N-terminal activation domain (NAD) and C-terminal domain (CAD) (Fig. 2D). Based on the above domains of NRF2, we constructed various truncation fragments of FLAG-tagged NRF2 and expressed them in endothelial cells. We found that the Neh2 domain (1-605aa) of NRF2 interacts with HA-HIF-2α, and when the Neh2 domain (1-86aa) is deleted, the interaction between other truncations of NRF2 and HA-HIF-2α disappears (Fig. 5E), suggesting that NRF2 interacts with HIF-2α via its Neh2 domain.

To determine the precise interaction mode between NRF2 and HIF-2α, we used ZDOCK to predict and verify the docking mode of the NRF2 Neh2 domain and HIF-2α in the nucleus. The Z-score results listed the most likely docking locations. Among the top 15 docking structures analyzed, the fifth-ranked structure had a highly complementary structure, suggesting that it is the docking mode of NRF2/HIF-2α.

Analysis of the docking structure revealed an intriguing interaction pattern between the DLG motif within the Neh2 domain of NRF2 and the PAS-B domain of HIF-2α, resembling a plug-and-socket configuration. This structural interplay was mainly due to electrostatic attractions between certain pairs of amino acid residues, particularly HIF-2α-R171 and NRF2-D29, HIF-2α-H234 and NRF2-E35, as well as HIF-2α-R179 and NRF2-D38. Furthermore, the electrostatic bonds involving NRF2-R34 and NRF2-D18/D21 facilitated the folding of the DLG sequence in NRF2, resulting in the formation of a stable plug-like assembly. Complementarily, the PAS-B domain of HIF-2α exhibited a concave, groove-like structure, highlighting the complexity of this molecular interaction (Fig. 5F).

The Adaptive Poisson-Boltzmann Solver (APBS) technique was used to image the electrostatic potential distribution on the surfaces of NRF2-Neh2 and HIF-2α-PAS-B proteins. The surface of NRF2-Neh2, characterized by a plug-like morphology, showed a distribution of negatively charged electrostatic potentials. Conversely, the groove-like feature within the HIF-2α-PAS-B internal cavity showcased a distribution of positively charged electrostatic potentials. This detailed mapping of the protein surface electrostatic potentials further demonstrates that the electrostatic interactions between NRF2-Neh2 and HIF-2α-PAS-B underpin the stability of their plug-and-socket configuration (Fig. 5G).

Given the importance of electrostatic interactions in the docking process between NRF2-Neh2 and HIF-2α-PAS-B, amino acid sequence alignment was performed across different species. This analysis aimed to pinpoint the preservation of positively and negatively charged amino acids within the docking interface of both NRF2-Neh2 and HIF-2α-PAS-B. The results of this analysis showed a significant degree of conservation of these charged amino acids across species, highlighting the universal nature of this regulatory mechanism (Fig. 5H).

To precisely assess the energetic contributions of residues at the interface between HIF-2α-PAS-B and NRF2-Neh2, docking and energy analyzes were performed to determine the binding of NRF2-Neh2 with the unaltered HIF-2α (S1), and with its mutants, including HIF-2α-R171A (S2), HIF-2α-R179A (S3), HIF-2α-H234A (S4), and the combined mutant HIF-2α-R171A/H234A/R179A (S5). Mutation of arginine (R) or histidine (H) to alanine (A) results in the elimination of the positive charge on these specific residues. After establishing the five binding models, we used the Molecular Mechanics/Generalized Born Surface Area (MM/GBSA) method to determine the binding free energy (ΔG_total) for each model, to quantify their respective contributions to the interaction. Among them, the S5-NRF2 model had the highest binding free energy, at -43.82 kcal/mol, while the binding free energies of the remaining models decreased sequentially from S3-NRF2 to S2-NRF2, S4-NRF2, and finally S1-NRF2. A comparative analysis of the binding models revealed that changes in the electrostatic interaction energy (ΔE_ele) were particularly significant, whereas the van der Waals interaction energy (ΔE_vdw) showed minimal variability. Furthermore, our protein interaction analysis revealed a collective contribution of positions R171, R179, and H234 on HIF-2α to the electrostatic interactions with NRF2-Neh2, among these residues R179 on HIF-2α providing the largest energy contribution, followed by HIF-2α-R171 and HIF-2α-H234 (Fig. 5I).

Building on the initial discovery of potentially interacting amino acid pairs (see Fig. 5F), we performed energy decomposition analysis on five docking models. This detailed analysis showed that amino acids involved in electrostatic interactions make a greater contribution to the energy landscape than those involved in van der Waals interactions. In particular, in the S1-NRF2 model, it was observed that the pair-forming amino acids were actively involved in electrostatic interactions. In the S2-NRF2 model, mutation of HIF-2α at R171A significantly reduced its electrostatic interaction with NRF2-D29. Similarly, mutation of HIF-2α to R179A in the S3-NRF2 model significantly reduced the electrostatic interaction with NRF2-E35/D38. Mutation to HIF-2α-H234A in the S4-NRF2 model resulted in a slight reduction in its electrostatic interaction with NRF2-E35. When the HIF-2α-R171A/H234A/R179A triple mutation was introduced into the S5-NRF2 model, it significantly enhanced the electrostatic repulsion against NRF2-D29/E35/D38. These results further support the notion that electrostatic attraction between critical amino acids in NRF2-Neh2 and HIF-2α-PAS-B is fundamental for their interaction, as shown in Fig. 5J.

Our investigation continued with the expression of HA-tagged versions of HIF-2α-WT and the HIF-2α-R171A/H234A/R179A mutant in diabetic endothelial cells. Nuclear proteins were extracted to evaluate the interaction between these proteins and NRF2 by co-IP. The results showed that the HIF-2α-R171A/H234A/R179A mutation significantly weakened its interaction with NRF2 in HUVECs under high glucose (25 mM) conditions, as shown in Fig. 5K. This finding reinforces the concept that the electrostatic attraction between NRF2-Neh2 and HIF-2α-PAS-B in diabetic endothelial cells plays a crucial role in their nuclear localization and retention.

**Figure 6:**
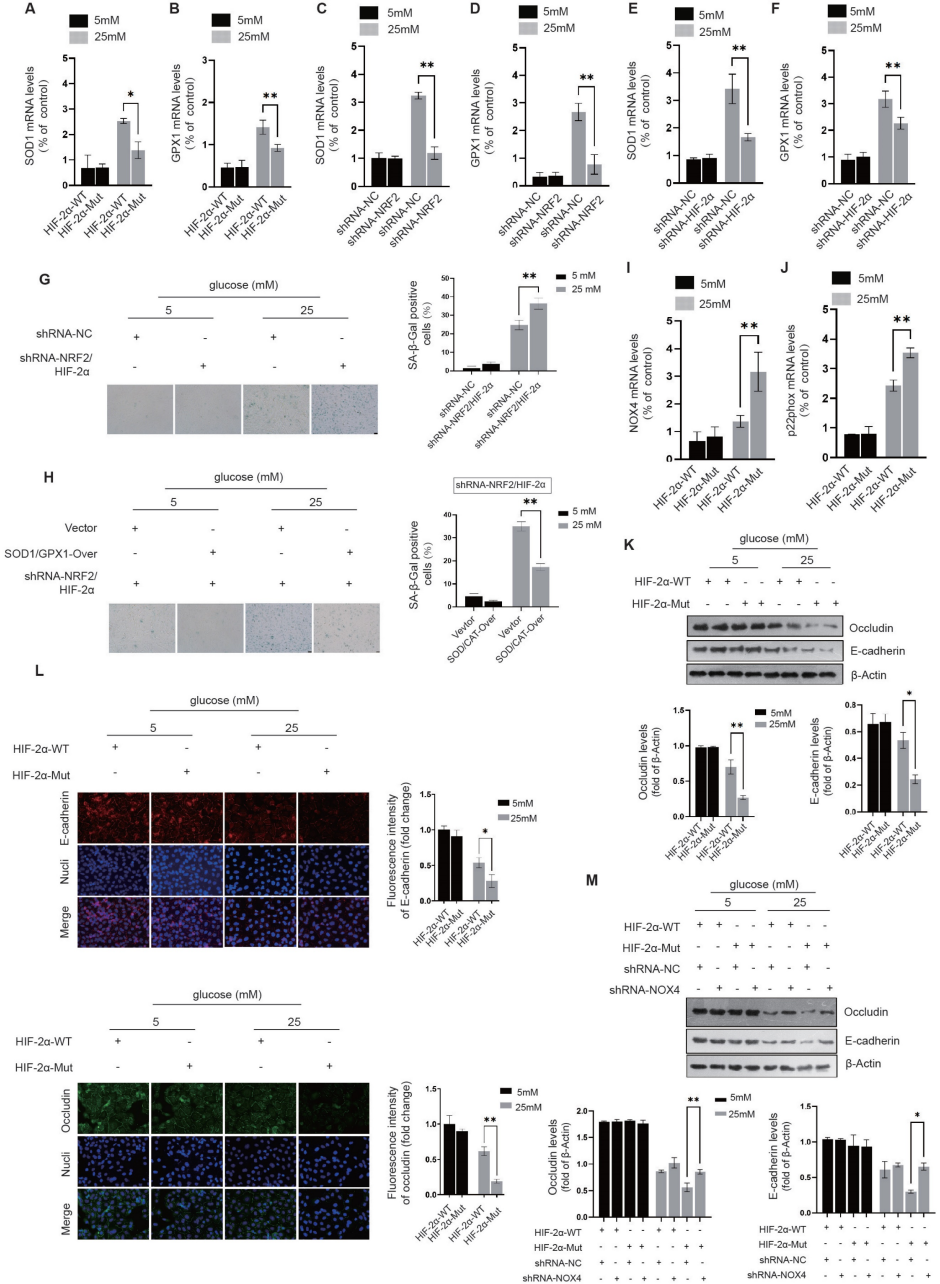
** NRF2 and HIF-2α act synergistically to alleviate cellular senescence and maintain intercellular junctions. A&B,** Following the silencing of endogenous HIF-2α, HUVECs were transfected with either exogenous HA-HIF-2α-WT or HA-HIF-2α-Mut (R171A/H234A/R179A) at glucose concentrations of 5 mM and 25 mM. The expressions of SOD1 and GPX1 were quantified using RT-qPCR. **C-F,** HUVECs were transfected with shRNA targeting either NRF2 (C&D) or HIF-2α (E&F) at glucose concentrations of 5 mM and 25 mM, and the expressions of SOD1 and GPX1 were analyzed using RT-qPCR. **G,** β-Galactosidase activity was measured in HUVECs after transfection with shRNA against NRF2 or HIF-2α at glucose levels of 5 mM and 25 mM. **H,** Upon overexpression of SOD1/GPX1 and simultaneous silencing of NRF2/HIF-2α in HUVECs at glucose concentrations of 5 mM and 25 mM, β-galactosidase activity was assessed. **I&J,** Following the protocol outlined in A&B, the expressions of NOX4 and p22phox in HUVECs were determined by RT-qPCR. **K&L,** In HUVECs treated as per A&B, the protein levels and membrane localization of occludin and E-cadherin were evaluated using Western blot and immunofluorescence staining, respectively. **M,** HUVECs were treated as described in A&B, with NOX4 expression further reduced using shRNA. The protein levels of occludin and E-cadherin were determined by Western blot.

**NRF2 and HIF-2α synergistically reduce mitochondrial oxidative stress in diabetic endothelial cells to alleviate cellular senescence.** By suppressing endogenous HIF-2α expression and introducing exogenous HA-HIF-2α-WT and HA-HIF-2α-Mut (R171A/H234A/R179A) mutants, we found a significant increase in the expression of SOD1 and GPX1 in the HA-HIF-2α-WT group under high glucose (25 mM) conditions. In contrast, in the HA-HIF-2α-Mut group, the expressions of SOD1 and GPX1 decreased significantly, indicating the essential role of NRF2-Neh2 and HIF-2α-PAS-B interaction in regulating the expression of these enzymes (Fig. 6A&B). Further investigation into the individual contributions of NRF2 and HIF-2α was performed by silencing either NRF2 or HIF-2α in HUVECs. This resulted in a significant decrease in upregulation of SOD1 and GPX1 expression under 25 mM glucose exposure, highlighting the cooperative function of NRF2 and HIF-2α in increasing the expression of SOD1 and GPX1 in diabetic endothelial cells (Fig. 6C-F).

To investigate how activation of NRF2 and HIF-2α influences cellular senescence in diabetic endothelial cells, both NRF2 and HIF-2α were knocked down, and the activity of β-galactosidase was measured. Knockdown of NRF2 and HIF-2α resulted in significant senescence in HUVECs under 25 mM glucose conditions (Fig. 6G). Furthermore, simultaneous overexpression of SOD1 and GPX1 together with knockdown of NRF2 and HIF-2α significantly attenuated the increase in β-galactosidase activity triggered by loss of NRF2/HIF-2α (Fig. 6H). These results suggest that concerted activation of NRF2 and HIF-2α in diabetic endothelial cells synergistically increases the expression of SOD1 and GPX1, thereby contributing to the alleviation of cellular senescence.

**The interaction between NRF2 and HIF-2α in diabetic endothelial cells inhibits NADPH oxidase expression and maintains intercellular junctions.** We engineered HUVECs to express exogenous HA-HIF-2α-WT and HA-HIF-2α-Mut while repressing the expression of endogenous HIF-2α. Under high glucose conditions (25 mM), we found a significant increase in the expression of NOX4 and p22phox in the HA-HIF-2α-Mut group compared to the HA-HIF-2α-WT group (Fig. 6I&J). This finding suggests that the interaction between NRF2 and HIF-2α plays a crucial role in preventing the upregulation of NOX4 and p22phox expression.

Additionally, we analyzed the protein levels and membrane localization of occludin and E-cadherin in HUVECs under high glucose (25 mM) conditions. It was observed that the protein levels of occludin and E-cadherin were significantly reduced in the HA-HIF-2α-WT group and were further reduced in the HA-HIF-2α-Mut group (Fig. 6K).

Immunofluorescence staining also showed that compared with the HA-HIF-2α-WT group, the integrated intensity of occludin and E-cadherin at the cell membrane was significantly impaired in the HA-HIF-2α-Mut group under high glucose conditions (Fig. 6L). These results highlight the importance of NRF2 and HIF-2α interaction in maintaining the protein levels and membrane localization of occludin and E-cadherin in diabetic endothelial cells. After the introduction of HA-HIF-2α-WT or HA-HIF-2α-Mut into HUVECs and concomitant knocking down NOX4, a significant improvement in maintaining occludin and E-cadherin protein levels in the HA-HIF-2α-Mut group (Fig. 6M). This observation further supports that the interaction between NRF2 and HIF-2α contributes to the maintenance of stable occludin and E-cadherin levels, possibly through inhibition of NADPH oxidase.

**Figure 7:**
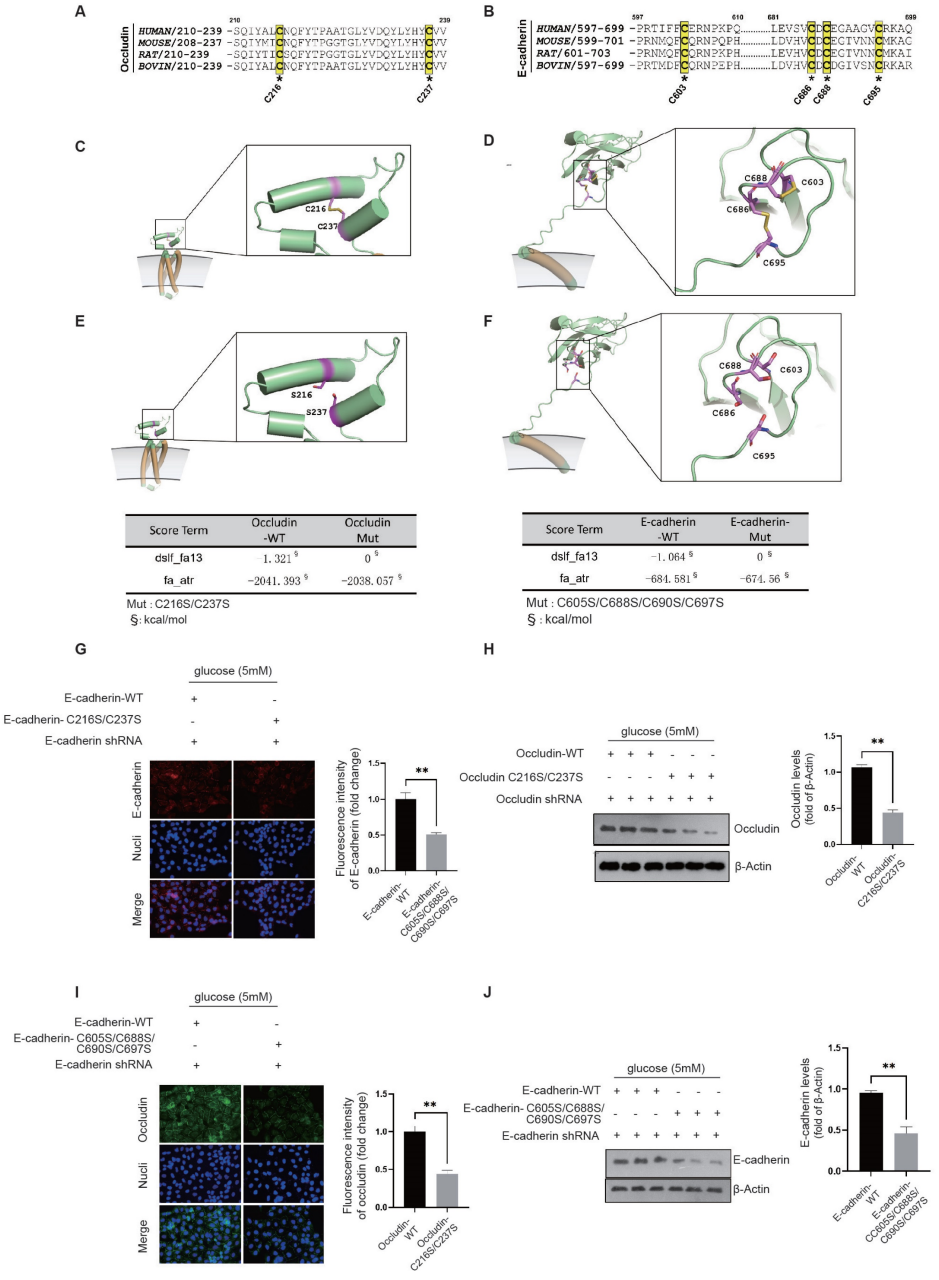
** NRF2/HIF-2α interaction inhibits NADPH-induced disability of occludin and E-cadherin structure to maintain intercellular junctions. A&B,** A conservation analysis was performed for cysteine residues on occludin and E-cadherin, which are crucial for the formation of disulfide bonds. **C&D,** The exact locations of these conserved disulfide bond pairs were pinpointed through the structural analysis of occludin and E-cadherin proteins. **E&F,** The impact of the disulfide bonds in occludin and E-cadherin was evaluated using Rosetta's SCORE algorithm to assess the force exerted by these bonds. **G&H,** HUVECs were transfected with either the occludin-WT or the occludin-C216S/C237S mutant, with endogenous occludin being silenced, at a glucose concentration of 5 mM. The membrane localization and protein levels of occludin were assessed through immunofluorescence staining and Western blot analysis, respectively. **I&J,** HUVECs underwent transfection with either the E-cadherin-WT or the E-cadherin-C603S/C686S/C688S/C695S mutant, while endogenous E-cadherin was silenced, at a glucose concentration of 5 mM. The membrane localization and protein levels of E-cadherin were determined via immunofluorescence staining and Western blot analysis, respectively.

Our structural analysis of the occludin protein revealed the presence of a pair of conserved cysteine residues, C216-C237, which form disulfide bonds (Fig. 7A). Similarly, in E-cadherin, we identified two pairs of conserved disulfide bonds across species, specifically C686-C695 and C603-C688 (Fig. 7B). Given that oxidative stress can compromise protein stability by disrupting disulfide bond formation, it is plausible to infer that NADPH oxidase plays a key role in the destabilization and degradation of occludin and E-cadherin in diabetic endothelial cells.

Occludin is characterized by its multiple transmembrane domains and plays an essential role in the establishment of intercellular junctions via its extracellular domains. The disulfide bond between C216-C237 is crucial for occludin's stability (Fig. 7C). Structural examination of the E-cadherin protein reveals two critical disulfide bonds, C686-C695 and C603-C688, located between its transmembrane domain and the domain that induces tight cell-cell junctions. Given the significant tension experienced by E-cadherin, the palindrome cross-structure formed by these two disulfide bond pairs evenly disperses the tension between cells by extending to the ends of the two-sheet secondary structures, thereby maintaining the stability of the E-cadherin structure (Fig. 7D). Using the SCORE algorithm in Rosetta software, we calculated the forces exerted by these disulfide bonds. The disulfide bond C216-C237 in occludin was found to contribute an energy of -1.321 kcal/mol (Fig. 7E). In E-cadherin, the disulfide bonds C603-C688 and C686-C695 were shown to contribute an energy of -1.064 kcal/mol (Fig. 7F). These energy decomposition analyses highlight the critical role of disulfide bond formation in the stability of E-cadherin and occludin.

The location and activity of NADPH oxidase near the cell membrane have a significant impact on oxidative stress in this area. We propose that the oxidative stress generated by NADPH oxidase in diabetic endothelial cells could lead to the breakdown of disulfide bonds in occludin and E-cadherin. Such disruption could lead to structural instability and make these proteins susceptible to degradation by matrix metalloproteinases in the extracellular matrix. To examine the effects of disulfide bond disruption on occludin and E-cadherin, we constructed occludin-C216S/C237S and E-cadherin-C603S/C686S/C688S/C695S mutants that replaced the original cysteine residues with serine. Notably, even at normal glucose concentration (5 mM), these mutants showed significantly reduced cell membrane localization and protein levels compared to their wild-type counterparts, as shown for occludin-C216S/C237S in Fig. 7G&H and for E-cadherin-C603S/C686S/C688S/C695S in Fig. 7I&J. These results demonstrate that the stability and integrity of occludin and E-cadherin critically depend on their disulfide bonds. The destruction of these bonds leads to reduced stability and increased degradation of the proteins, thereby compromising intercellular junctions. This highlights the protective role of the NRF2 and HIF-2α interaction, which appears to attenuate the action of NADPH oxidase and thereby preserve the structural integrity of occludin and E-cadherin by keeping their disulfide bonds intact.

**Oltipraz attenuates cellular senescence while preserving intercellular junctions in diabetic HUVECs.** Our preliminary results showed that in HUVECs exposed to mild (10 mM) to moderate (15 mM) glucose concentrations, acetylation-dependent activation of NRF2, coupled with its nuclear interaction with HIF-2α, plays a critical role in attenuating cellular senescence and the maintenance of intercellular junctions. However, this protective mechanism fails under high glucose conditions (25 mM), indicating an urgent need to develop effective small molecule therapeutics capable of effectively increasing NRF2 activation while simultaneously inhibiting HIF-2α activity to protect endothelial cells. Previous studies have identified several NRF2 activators, including Sulforaphane, Dimethyl fumarate, Bardoxolone methyl, Oltipraz, Curcumin, and EGCG. Among these, Oltipraz stands out for its additional inhibitory effect on HIF-1α, positioning it as a promising candidate for effective therapeutic intervention.

**Figure 8:**
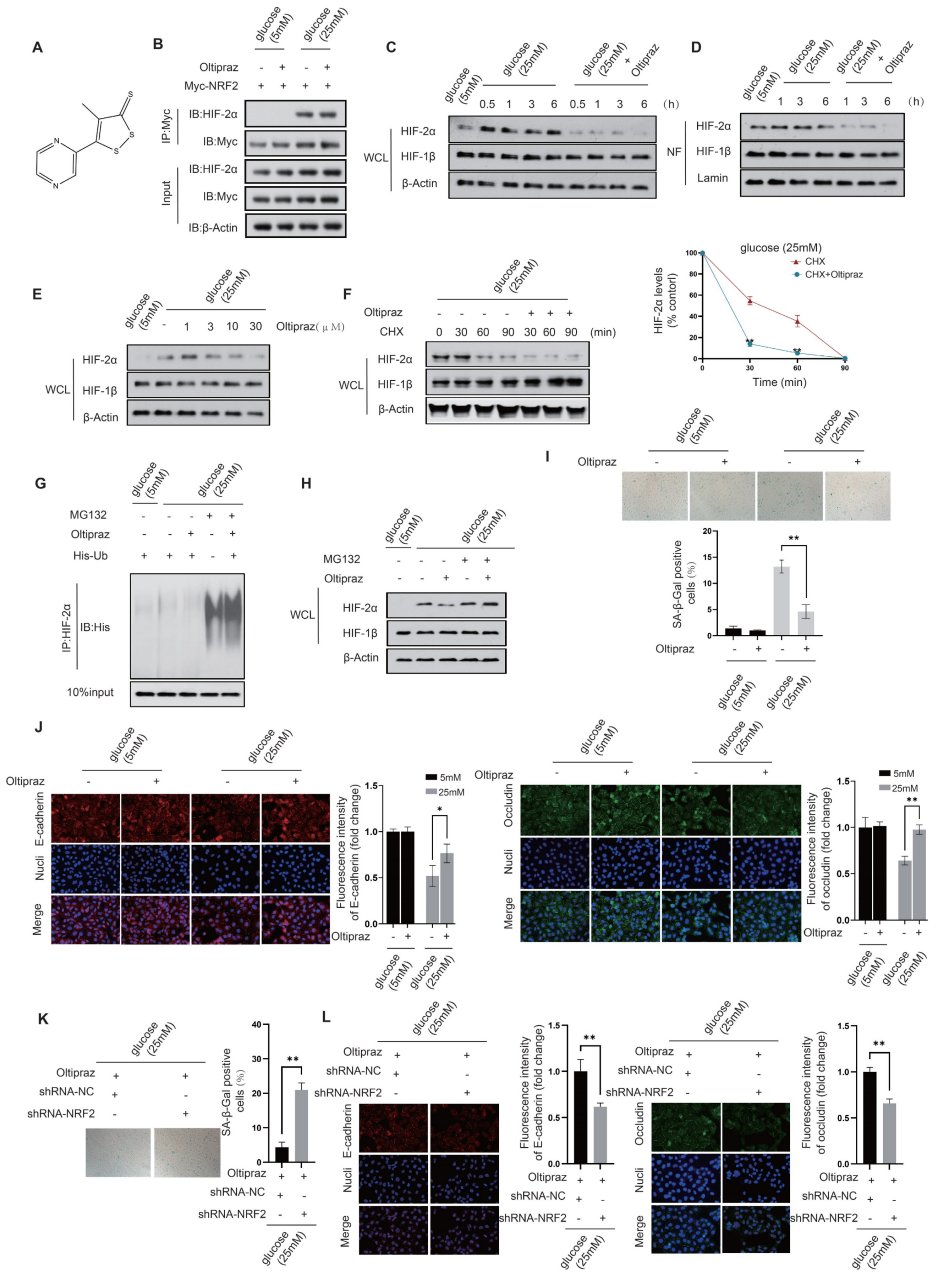
** Oltipraz attenuates cellular senescence while preserving intercellular junctions in diabetic HUVECs. A.** Molecular structure of Oltipraz.** B.** The NRF2-HIF-2α interaction in Oltipraz-treated HUVECs was identified using a co-IP assay. **C&D.** Western blot analysis was used to assess the protein levels of HIF-1β and HIF-2α in the nuclear fraction (NF) (D) and the whole-cell lysate (WCL) (C) of HUVECs treated with Oltipraz. **E.** The protein levels of HIF-1β and HIF-2α in HUVECs exposed to various concentrations of Oltipraz were determined through Western blot analysis. **F.** Following treatment with cycloheximide (CHX), the variations in HIF-1β and HIF-2α protein levels in Oltipraz-treated HUVECs were monitored over different time intervals. The right panel illustrates the temporal pattern of HIF-2α protein level changes across the groups. **G&H.** The effect of Oltipraz on the ubiquitination (G) and protein levels (H) of HIF-2α was examined in HUVECs after MG132 treatment. **I.** The β-galactosidase activities in HUVECs treated with Oltipraz were measured at glucose concentrations of 5 mM and 25 mM. **J.** The localization of occludin and E-cadherin in Oltipraz-treated HUVECs was evaluated using immunofluorescence at glucose concentrations of 5 mM and 25 mM. **K.** β-Galactosidase activity was assessed in HUVECs after transfection with shRNA targeting NRF2, under Oltipraz treatment, at a glucose concentration of 25 mM. **L.** In HUVECs treated as described in K, the subcellular localization of occludin and E-cadherin was evaluated using immunofluorescence staining.

We next investigated the regulatory effects of Oltipraz on NRF2 and HIF-2α (Fig. 8A). Co-IP experiments revealed that Oltipraz did not significantly alter the interaction between NRF2 and HIF-2α in HUVECs exposed to a high glucose (25 mM) condition, (Fig. 8B). This observation suggests that Oltipraz could directly activate NRF2 and simultaneously inhibit HIF-2α activation, thereby producing a protective effect on endothelial cells.

After Oltipraz treatment, we observed a gradual decrease in HIF-2α protein levels in both the nuclear fraction and total cell lysate of HUVECs exposed to a high glucose (25 mM) environment (Fig. 8C&D). This trend suggests a suppressive effect of Oltipraz on HIF-2α. Further investigation revealed that Oltipraz treatment resulted in a concentration-dependent reduction in HIF-2α protein levels, with the most significant suppression observed at 30 μM (Fig. 8E). Oltipraz significantly increased the rate of HIF-2α degradation in the presence of a high glucose concentration (25 mM) when protein synthesis was blocked with cycloheximide (CHX) (Fig. 8F). This provides additional evidence for a likely association with increased HIF-2α ubiquitination (Fig. 8G&H).

Experimental validation showed that Oltipraz treatment under high glucose (25 mM) conditions significantly reduced β-galactosidase activity in HUVECs (Fig. 8I). Furthermore, Oltipraz effectively counteracted the reduction in fluorescence intensity of key intercellular junction proteins, occludin and E-cadherin (Fig. 8J). These results indicate that Oltipraz plays an important role in alleviating cellular senescence and maintaining the integrity of intercellular junction in HUVECs exposed to high glucose conditions. Consequently, Oltipraz holds promise as a pharmacological strategy for treating complications arising from vascular endothelial damage in diabetes. In the context of Oltipraz intervention, we further downregulated NRF2 to validate the role of Oltipraz in simultaneously regulating endothelial cell senescence and intercellular junctions via the NRF2-HIF2α signaling pathway in diabetes. Oltipraz significantly alleviated endothelial cell senescence and maintained intercellular junctions in a 25mM glucose environment, whereas these protective effects disappeared concomitantly with NRF2 knockdown (Fig. 8K&L). This finding further confirms the regulatory role of Oltipraz based on NRF2-HIF2α signaling.

## Discussion

In the context of diabetes, when endothelial cells enter a state of senescence, there is a simultaneous deterioration of intercellular junctions. However, the exact mechanisms driving this process are not yet fully understood from previous studies. Our study revealed that under conditions of mild to moderate glucose elevation, endothelial cells initiate inherent defense responses aimed at combating cellular senescence and preserving the integrity of intercellular junctions. These protective mechanisms involve i) The acetylation-dependent activation of NRF2, which enhances the production of cytoplasmic antioxidant proteins, effectively counteracting cellular senescence. ii) The interaction between NRF2 and HIF-2α suppresses HIF-2α-induced NADPH oxidase expression, thereby contributing to the maintenance of intercellular junctions by reducing the disruption of disulfide bonds within occludin and E-cadherin. Although these intrinsic defenses remain active under high glucose conditions, they prove insufficient to fully attenuate the oxidative stress reactions in the cytoplasm and at the cell membrane. As a result, the simultaneous occurrence of endothelial cell senescence and deterioration of intercellular junctions is observed.

Hence, the development of therapeutic drugs capable of stimulating NRF2 while concurrently inhibiting HIF-2α signaling is crucial. In our study, we focused on evaluating well-documented NRF2 activators, including Sulforaphane, Dimethyl fumarate, Bardoxolone methyl, Oltipraz, Curcumin, and EGCG. In particular, oltipraz has shown promise in assisting endothelial cells to overcome cellular senescence and maintain the integrity of intercellular junction under high glucose (25 mM) conditions. By simultaneously modulating the NRF2 and HIF-2α signaling pathways, Oltipraz bolsters the inherent defense mechanisms of endothelial cells. This dual action allows endothelial cells to effectively resist senescence and maintain intercellular junctions, providing a potential therapeutic advantage for patients with hyperglycemia.

Our research has found that as diabetes progresses, endothelial cells show signs of senescence and impaired intercellular junctions. These changes contribute to endothelial dysfunction and increased vascular permeability, rendering the vessel walls more susceptible to damage and inflammation.

Consequently, this situation fosters the onset of atherosclerosis and valvular calcification. Solely addressing endothelial cell senescence or disruption of intercellular connections may not be sufficient to halt disease progression. Therefore, a therapeutic strategy using small molecule drugs to target these two pathological processes simultaneously could provide a more effective way to prevent the progression of these diseases.

The employment of senolytic drugs in eliminating senescent cells has exhibited advancements in vascular function and a reduction in inflammatory processes in preclinical diabetes models (Baker *et al.*, 2016). Furthermore, molecules that stabilize endothelial junctions have displayed promising results in mitigating vascular permeability and inflammation (Garcia *et al.*, 2018). Nevertheless, these medications solely exhibit a single therapeutic effect, insufficient in addressing the multifaceted damage inflicted upon endothelial cells by diabetes. Recent studies have underscored the efficacy of small-molecule drugs in tackling complex pathologies by simultaneously modulating multiple pathways (Smith *et al.*, 2020). The development and clinical evaluation of such multi-action small molecule drugs could pave the way for innovative treatments that significantly enhance cardiovascular outcomes among diabetic patients. This approach underscores the significance of translating basic research findings into practical, clinically applicable therapies that cater to the multifaceted nature of diabetes and its associated complications.

Previous research has shown that anti-senescence compounds, particularly rapamycin, can delay endothelial cell senescence and mitigate vascular damage. Drugs such as ACE inhibitors and ARBs are known to maintain the integrity of the vascular endothelial barrier by regulating the expression of intercellular junction proteins. Nonetheless, these drugs cannot simultaneously combat endothelial cell senescence and disruption of intercellular junctions, which limits their clinical effectiveness. Oltipraz, with its unique ability to simultaneously modulate both endothelial cell senescence and disruption of intercellular junctions, is emerging as a promising therapeutic approach for the treatment of atherosclerosis and valvular calcification in diabetic patients.

## Materials and methods

**Cell culture.** Human umbilical vein endothelial cells (HUVECs) were cultured in Dulbecco's Modified Eagle's Medium (DMEM) supplemented with 10% fetal bovine serum (FBS), 2 mM L-glutamine, 100 IU/mL penicillin, and 100 μg/mL streptomycin. The cells were kept in a 37°C humidified incubator with 5% CO2.

**Generation of gene-knockdown and put-back HUVECs.** To inhibit the expression of these endogenous genes, lentiviruses containing shRNA targeting human NRF2, HIF-2α, occludin, and E-cadherin (RepoBio, Hangzhou, China) were transfected into HUVECs. To reintroduce different exogenous genes, lentivirus plasmids encoding wild-type and mutant versions of genes such as NRF2, HIF-2α, occludin, and E-cadherin (RepoBio, Hangzhou, China), which are resistant to shRNA, were constructed and used for transfection.

**β-galactosidase activity assay.** Senescent cells were detected using the β-galactosidase activity assay. Briefly, cells were washed with phosphate-buffered saline and fixed with 4% paraformaldehyde for 10 min. Cells were then washed again with phosphate-buffered saline and incubated with β-galactosidase staining solution containing 5-bromo-4-chloro-3-indolyl-β-D-galactopyranoside (X-gal) overnight at 37°C. Finally, the cells were observed under a microscope and the percentage of senescent cells was calculated based on the number of blue-stained cells.

**CellROX Orange Reagent staining.** Intracellular ROS was measured using CellROX® Orange Reagent (Thermo Fisher Scientific, Waltham, MA, USA) and flow cytometry analysis. HUVECs were seeded in a 6-well plate and treated with drugs for 24 h. After treatment, cells were washed with PBS and incubated with CellROX Orange Reagent (5 μM) for 30 min at 37°C in the dark. Cells were then washed twice with PBS, harvested by trypsinization, and resuspended in PBS for flow cytometry analysis. CellROX fluorescence intensity was measured using a flow cytometer (BD FACSCanto II) and analyzed using FlowJo software.

**MitoSOX staining.** HUVECs were seeded in 6-well plates and allowed to grow for 24 hours. MitoSOX (Invitrogen) was diluted in serum-free medium to a final concentration of 5 μM and added to each well. The cells were then incubated at 37 °C for 30 min. After staining, cells were washed with PBS and harvested with trypsin. Cells were then resuspended in 500 μL ice-cold PBS and subjected to flow cytometry analysis using a FITC channel.

**Co-immunoprecipitation.** Cells were lysed with lysis buffer containing a protease inhibitor cocktail and a phosphatase inhibitor cocktail for 30 min on ice. The lysate was then centrifuged and the supernatant was collected. For co-immunoprecipitation, 500 μg of total protein was incubated with primary antibody and protein A/G beads overnight at 4°C. The beads were then washed and the eluted proteins were analyzed using Western blot.

**Western blot analysis.** HUVECs were treated with drugs for 24 h, lysed in RIPA buffer, and protein was measured using a BCA assay kit. Equal amounts of protein (20 μg) were separated by SDS-PAGE and transferred onto PVDF membranes. Primary antibodies against target proteins were incubated overnight at 4°C, followed by secondary antibody incubation for 1 h. Proteins were visualized using ECL detection and analyzed using ImageJ software.

**Quantitative RT-PCR.** Quantitative RT-PCR was performed using PowerUp SYBR Green Master Mix (Thermo Fisher Scientific, Waltham, MA, USA) on a StepOnePlus Real-Time PCR system (Thermo Fisher Scientific, Waltham, MA, USA). The primers for target genes and reference gene (GAPDH) were designed using Primer-BLAST (NCBI) and synthesized by Invitrogen (Thermo Fisher Scientific, Waltham, MA, USA). The cycling conditions were as follows: 50°C for 2 min, 95°C for 2 min, and 40 cycles of 95°C for 15 s and 60°C for 1 min. The relative mRNA expression was calculated using the 2^-ΔΔCT method.

**ZDOCK for analysis of NRF2 and HIF-2α interaction.** The protein docking analysis was performed using ZDOCK, a widely used software program for protein docking. The 3D structures of the proteins of interest were obtained from the Protein Data Bank (PDB) and used as inputs for the ZDOCK analysis. The docking protocol consisted of three main steps: (1) pre-processing of the protein structures to remove water molecules and non-protein atoms; (2) binding site identification and assignment of grids for both proteins; and (3) docking of the two proteins using a fast Fourier transform (FFT) algorithm. ZDOCK analysis generates a plethora of potential complex structures through a fast Fourier transform-based algorithm. These structures are evaluated using the ZDOCK scoring function, which includes factors such as electrostatics, van der Waals forces, and solvation energy. The scoring algorithm allowed the resulting structures of NRF2/HIF-2α complexes to be ranked for co-IP verification.

**Molecular Mechanics/Generalized Born Surface Area (MM/GBSA) for calculating binding energy.** Molecular Mechanics/Generalized Born Surface Area (MM/GBSA), have been employed to estimate protein-protein binding free energies. When calculating the binding free energy in protein-protein interactions using MM/GBSA, several contributions are considered. The total binding free energy is represented by ΔGbind, while the total gas-phase energy is represented by ΔEMM, which includes the internal energy from bond, angle, and dihedral terms in the MM force field (ΔEinternal), electrostatic energy (ΔEelectrostatic), and van der Waals energy (ΔEvdw). The polar contribution to solvation-free energy (ΔGPB/GB) and the nonpolar solvation-free energy (ΔGSA), which is usually estimated by a linear function of solvent-accessible surface area (SASA), are also considered. The binding-induced conformational entropy (-TΔS) is estimated through normal-mode analysis. Note that ΔEinternal is always zero in MM/PBSA and MM/GBSA calculations that are based on a single complex trajectory. The polar contribution to solvation-free energy (ΔGPB/GB) is estimated with either the Poisson-Boltzmann (PB) or generalized Born (GB) method, while ΔGSA is estimated using a linear function of SASA. The binding free energy for a protein-protein complex can be calculated using the following equations:



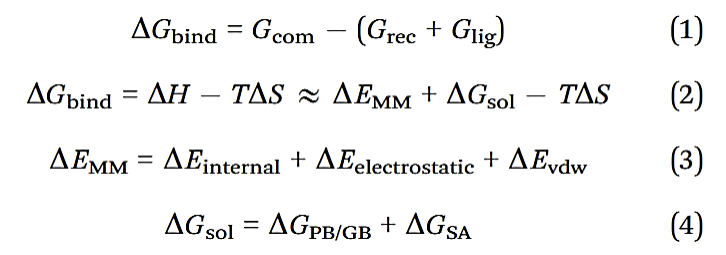



**Statistical analysis.** Data are presented as the mean ± standard deviation (SD) of at least three independent experiments. Statistical significance was determined by one-way ANOVA followed by Tukey's post-hoc test using GraphPad Prism version 8.0 (GraphPad Software Inc., San Diego, CA, USA). P values less than 0.05 were considered significant.
